# Barriers and facilitators to implementing a patient-centered model of contraceptive provision in community health centers

**DOI:** 10.1186/s40834-016-0032-3

**Published:** 2016-11-08

**Authors:** Mary C. Politi, Amy Estlund, Anne Milne, Christina M. Buckel, Jeffrey F. Peipert, Tessa Madden

**Affiliations:** 1 Division of Public Health Sciences, Department of Surgery, Washington University in St. Louis School of Medicine, 660 S Euclid Ave, CB 8100, St Louis, MO 63110 USA; 2 Divisions of Family Planning and Clinical Research, Department of Obstetrics & Gynecology, Washington University in St. Louis School of Medicine, 4533 Clayton Ave, Box 8019, St. Louis, USA; 3 Department of Obstetrics & Gynecology, Indiana University School of Medicine, 550 N. University Blvd, UH 2440 Indianapolis, USA

**Keywords:** Contraception, Qualitative research methods, Providers, Quality of care

## Abstract

**Background:**

The Contraceptive CHOICE Project developed a patient-centered model for contraceptive provision including: (1) structured, evidence-based counseling; (2) staff and health care provider education; and (3) removal of barriers such as cost and multiple appointments to initiate contraception. In preparation for conducting a research study of the CHOICE model in three community health settings, we sought to identify potential barriers and facilitators to implementation.

**Methods:**

Using a semi-structured interview guide guided by a framework of implementation research, we conducted 31 qualitative interviews with female patients, staff, and health care providers assessing attitudes, beliefs, and barriers to receiving contraception. We also asked about current contraceptive provision and explored organizational practices relevant to implementing the CHOICE model. We used a grounded theory approach to identify major themes.

**Results:**

Many participants felt that current contraceptive provision could be improved by the CHOICE model. Potential facilitators included agreement about the necessity for improved contraceptive knowledge among patients and staff; importance of patient-centered contraceptive counseling; and benefits to same-day insertion of long-acting reversible contraception (LARC). Potential barriers included misconceptions about contraception held by staff and providers; resistance to new practices; costs associated with LARC; and scheduling challenges required for same-day insertion of LARC.

**Conclusions:**

In addition to staff and provider training, implementing a patient-centered model of contraceptive provision needs to be supplemented by strategies to manage patient and system-level barriers. Community health center staff, providers, and patients support patient-centered contraceptive counseling to improve contraception provision if organizations can address these barriers.

## Background

Unintended pregnancy is a persistent public health problem in the United States, with almost half of the 6.1 million pregnancies that occur annually in the U.S. being unwanted or mistimed [1]. Unintended pregnancy has been associated with adverse socioeconomic and health outcomes for women and their children [2]. Use of contraception significantly reduces unintended pregnancies and births and improves maternal and infant health outcomes [2]. However, multiple barriers limit women’s access to and use of effective contraception. For example, out-of-pocket costs can limit women’s access to and appropriate use of many contraceptive methods [3, 4]; Patient out-of-pocket costs have only been partially addressed by the Affordable Care Act (ACA) due to challenges in implementation [5]. In addition, clinician misconceptions may limit the use of highly effective methods such as intrauterine devices (IUDs) and implants among some women, especially among adolescents, nulliparous women, and women with a history of sexually transmitted infections [6–8]. Requirements for patients to return for a second visit for placement of an intrauterine device (IUD) or contraceptive implant can decrease the likelihood of initiation of these methods [9, 10]. Lack of post-visit contraceptive support further contributes to contraception non-adherence [11].

The Contraceptive CHOICE Project (CHOICE) was a longitudinal, observational study of 9,256 women provided with no-cost contraception to reduce barriers to long acting reversible methods [12]. In addition to removing financial barriers, CHOICE developed a patient-centered model for comprehensive contraceptive provision that responded to patients’ individual preferences and values and guided by the IOM definition [13], including (1) structured, evidence-based contraceptive counseling to improve patients’ contraception knowledge [14]; (2) staff and provider education about reversible contraceptive methods; and (3) the removal of system- level barriers such as cost and return appointments to the clinic to initiate the preferred contraceptive method. The CHOICE counseling was based on the GATHER framework [15]. Providing contraception using this model in a research setting led to an increased uptake of IUDs and implants [12], higher rates of contraceptive satisfaction and continuation, and significant reductions in unintended pregnancy and birth [12, 16]. A similar model of care implemented in Colorado removed barriers by providing no-cost lUDs and implants to women and training health center staff and providers at Title X clinics. Implementation of the state-wide program resulted in an increase in IUD and implant use and a reduction in high-risk births and abortions [17].

**Fig. 1:**
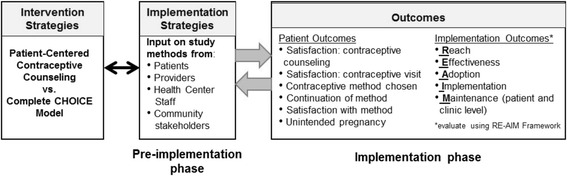
Implementation Process Framework Guiding The Study

Despite evidence of effectiveness, the CHOICE model has not yet been implemented in clinical practice on a large scale, nor has there been any comparison of the effect of the counseling versus the other components of the model. According to one framework for implementation research (Fig. 1), adopting evidence-based practices in routine care requires the engagement of multiple stakeholder groups. In addition, researchers need to measure and explore both patient-level outcomes as well as implementation process outcomes to ensure successful adoption and dissemination of interventions in real world settings. Based on this model, the “Innovative Model of PAtient-Centered ContracepTion (IMPACCT)” study was designed to test the effectiveness of the CHOICE contraceptive counseling compared to the full CHOICE model in three community health centers. In this manuscript we describe the results of stakeholder interviews with patients, staff, and healthcare providers to identify barriers and facilitators to implementing the CHOICE components in practice, following the first step in the implementation process model.

## Methods

### Study design

This study was conducted between October 2013 and January 2014 at two Federally Qualified Health Centers (FQHCs) in St. Louis, MO and one FQHC in Memphis, TN. These health centers serve predominantly low-income, urban, and underserved populations. The research team recruited these three centers based on previous collaboration and research interests. Research and trained health center staff approached eligible female patients during their scheduled health center appointment, described the study, and invited them to participate. Prior to the recruitment of health center employees, the research team delivered in-person presentations for staff at each site, which described the research study. Health center staff and providers were then invited to participate in the study by clinic administrators at each site based on the staff members’ involvement in the provision of contraceptive care. Eligible participants included English-speaking female patients between the ages of 14 and 45 presenting to the clinic for a family planning service, health center staff (e.g., front desk assistants, patient service representatives, medical assistants, registered nurses, office managers), or health care providers from the three FQHCs.

Qualitative methods highlight personal values and practices that may welcome or hinder adoption or implementation of a new practice, explore others’ perspectives in depth, and provide insight into group issues of interest [18, 19]. Consistent with implementation research processes [20, 21], the research team conducted qualitative interviews with stakeholders to gain an understanding of whether and how the components of the CHOICE model could be adopted into clinical practice and to identify potential barriers prior to implementation.

### Data collection

We developed a semi-structured interview guide based on past work on this topic [22], adapting sample interview guides using Proctor’s framework of implementation research [Fig. 1] [21]. The framework suggests that preparation for implementation should include identification of an evidence-based strategy, exploration of the perception of the strategy among key implementation audiences, and evaluation of the strategy using patient-level and implementation-level outcomes. This study focused on exploring perceptions of the CHOICE counseling and other components of the CHOICE model.

The interview guide was structured to gather input on patient-, provider-, and system-level factors that may affect provision of contraceptive services and implementation of the two different models of care. Interviews with patients asked about attitudes, beliefs, perceived social norms, barriers and facilitators to receiving contraception, and barriers and facilitators to contraceptive continuation. Interviews with health center providers and staff asked about the organizational culture and philosophy of the center, practices related to contraception provision, and barriers and facilitators to providing appropriate contraception. The interviews were conducted by two members of the research team (TM and CB) trained in qualitative research methods. They inquired about available resources for contraceptive services, including existing materials used for patient education, the acceptability of providing structured contraceptive counseling and the CHOICE model, and health care provider readiness to implement new models of contraceptive care at the center. We continued interviews until thematic saturation was reached, reviewing transcripts periodically during data collection. Interviews were audio-recorded with participant permission and lasted between 20–40 min. Participants received a $25 gift card in appreciation of their time. Washington University in St. Louis Human Research Protection Office approved the study prior to any research procedures. Interviews were audiotaped, transcribed verbatim, and uploaded into NVivo 10 software for analysis [23].

### Analysis

Interviews were analyzed following a grounded theory approach. We used open coding, followed by axial coding, to analyze the interview transcripts. Open coding involves coding the data for main categories or themes where axial coding allows for further investigation into core findings [24]. Two masters’ level members of the research team (AM and AE), trained in qualitative research methods, independently coded transcripts. The research team met to review coding and resolve discrepancies by consensus. We then recoded the transcripts and calculated inter-rater reliability (IRR) with a mean Kappa of 0.93 and mean percent agreement of 99.7 % (range 83.29–100 %) for ten double-coded transcripts. Each coder also independently coded three additional transcripts, which were then reviewed by the other coder to ensure adherence to the codebook. Having reached coding agreement, the remaining 18 transcripts were divided between coders. Coders brought any questions or discussion points about coding to the full team for review and resolution.

The research team met as a group to identify and discuss emerging themes related to patient-, provider-, and system-level factors that influence contraceptive practices and provision. The team identified quotes from transcripts that supported the main themes and provided an understanding of the barriers and facilitators to implementing this model of patient-centered contraceptive provision.

## Results

### Sample

We conducted in-person qualitative interviews with a total of 31 participants (13 patients and 18 health center staff and providers) from the three FQHCs. Five additional patients were approached for participation but declined due to time constraints. All clinic staff members who were approached participated in the study. Table 1 summarizes demographic characteristics of the sample. The majority of patient participants were black, non-Hispanic, aged 21–34, had at least some college, and were single and never married. Over half of the patients had a monthly income of less than $800. Most FQHC staff and provider participants were over the age of 34 and held positions involving some degree of clinical interaction with patients. Half of the staff and provider participants held a college degree or higher, while 45 % had some college or vocational training. Staff and provider participants averaged 15 years of experience in healthcare and 8 years of experience in family planning.

**Table 1:** Demographic characteristics of interviewees

	Patients, *n* = 13 N (%)	Staff and providers, *n* = 18 N (%)
Age		
≤ 20	2 (15.4)	0
21–29	5 (38.5)	2 (11.6)
30–39	5 (38.5)	8 (44.4)
≥ 40	1 (7.7)	8 (44.4)
Race		
Black	10 (76.9)	9 (50.0)
White	2 (15.4)	7 (38.9)
Ethnicity		
Hispanic	1 (7.7)	2 (11.1)
Insurance		
None	3 (23.1)	n/a
Public	6 (46.2)	n/a
Private	4 (30.8)	n/a
Education		
≤ high school	4 (30.8)	1 (5.6)
Some college/vocational	6 (46.2)	8 (44.4)
College +	3 (23.1)	9 (50.0)
Marital status		
Never married	9 (69.2)	n/a
Cohabitating	4 (30.8)	n/a
Monthly Income		
≤ $800	7 (53.9)	n/a
$801–$1600	3 (23.1)	n/a
≥ $1601	3 (23.1)	n/a
Patient history		
Years at participating center, M (SD)	10 ± 8.28	n/a
Employment History		
Years in healthcare	n/a	15.4 ± 12.0
Years in family planning	n/a	7.8 ± 7.3
Years at participating center	n/a	5.9 ± 5.1
Center role		
Front desk	n/a	3 (16.7)
Medical Assistant	n/a	5 (27.8)
RN or LPN	n/a	4 (22.2)
Provider	n/a	4 (22.2)
Management/Administration	n/a	2 (11.1)

### Overview

Three main themes emerged through the coding process. First, providers, staff, and patients identified significant misconceptions and knowledge gaps across all three stakeholder groups. They also described a lack of training that hindered provider and staff ability to provide comprehensive services. Second, participants indicated general support for the CHOICE model components, which include contraceptive counseling, same-day insertions, and staff training. Participants stated that these services would be beneficial to patients and the health center. Finally, interviewees acknowledged that in order to implement the CHOICE model, several barriers would have to be addressed at the system, provider/staff, and patient levels.

### Knowledge and training gaps among participants

Patients described misinformation and misconceptions about contraceptive methods and their effectiveness. In general, interviewees demonstrated a lack of knowledge about basic contraceptive information. One patient stated,

*“I think there’s something like a sponge…I don’t really know how that works but I’ve heard about it. I don’t know that much. I’ve heard the name [IUDs] but I still don’t know much about it.” - Patient*

Patients received both true and false information from a variety of sources, including family, friends, and providers. One patient specifically changed behavior based on information received from a doctor and became pregnant because of it:

*“…the doctor told me with me having a LEEP done, there was like a slim to none chance that I would ever get pregnant…so, I just got off birth control then.” - Patient*

Many patients indicated that friends and family heavily influence their contraceptive decision making.

*“[My mom] thinks I should get it [hormonal IUD]. She thinks it’s a great idea…she wanted to find something that’s more comfortable for me to get…my godmother had it and she told me it was good. So I was like “Well I should try it.” She was like “It slows up your bleeding. If you had heavy bleeding, you’ll like it. And you don’t get too fat, losing hair.” And I was like, well yeah, I should try it.” – Patient*

*“I talked to my mom about it…she said when she was growing up she used the pill. But that’s what made me try it.” - Patient*

While family and friends can be trusted sources of information, sometimes this casual information sharing can perpetuate fear and misinformation.

*“When I told her [my mom] I was going to get the [DMPA shot] she was pretty supportive. She just said that maybe I should take a break from it every now and then…you know that would make sense, to get my body back to its normal routine. That’s what I did now.” – Patient*

*“People think condoms are always going to work and they’re not. They’re using word of mouth of what other people say bad about birth control…about weight gain and blood clots…a lot of people fear stuff like that.” - Patient*

Additionally, several patients did not have a general understanding about risks, side effects, or effectiveness. One patient expressed suspicion toward IUDs because of what she perceived to be risks associated with this type of birth control:

*“IUDs can cause bacterial infections and stuff like that.” - Patient*

Staff and providers indicated they desired more training about specific contraceptive methods. This lack of training prevented them from providing comprehensive contraceptive education and services. While it is acknowledged that health center staff do not prescribe or insert contraception, several staff indicated they were not included in any contraceptive training.

*“[F]rom my perspective you don’t actually get like trained on specific birth control…the whole training thing is…knowing to like get the patients’ vitals and…set up for the pap smear, that type of thing. The training is not really directed towards like the different birth controls itself.” - Staff*

Another staff member admitted to not knowing basic contraceptive information about IUDs and implants, saying:

*“I get confused with like the [hormonal IUD], is it progesterone and estrogen and then the IUD – all that stuff. The [Implant]. That stuff kind of gets confusing to me so I don’t have that right information.” - Staff*

Additionally, some providers who were in the position to provide IUDs and implants indicated they were not trained to do so. Participants also acknowledged that training among their colleagues was limited. One provider spoke to both of these barriers when she said:

*“Well, I currently don’t even put [IUDs and implants] in now because I haven’t had the training…Right now I just provide the educational piece…” and she continued later to say, “I don’t think there is too many of them that are even certified.” - Provider*

### Support for the key components of the CHOICE model

Participants described overall support for the three main components of the CHOICE model. Several commented on the added benefit the model would provide. Patients, staff, and providers described the importance of personalized, comprehensive contraceptive counseling to facilitate informed choice. One patient identified a need for autonomy and acknowledgement of unique patient needs and values:

*“It’s important that the doctor just educates them [patients] as much as they can for the availability of all the different types because a lot of people are different and they want different things and not be pushed into a particular type.” - Patient*

Staff supported the concept of providing education and letting patients take time with the new information they receive. These participants wanted their patients to be empowered and informed. One staff said*:*

*“I think if we could bring them in, just do a little bit more educational [sic], not just in a room for 5–10 min, but maybe 30 min. Let them ask questions. Let them get the feel of everything…all of the birth controls that we offer.” - Staff*

Another commented:

*“I think education would be a great benefit to these young ladies around here. It really would. It would help out a whole lot because a lot of them just don’t know. They just don’t know.” - Staff*

In addition to the benefit of comprehensive contraceptive information, staff and providers felt that the option for same-day insertion of LARC methods could greatly benefit their patients. Several staff and providers acknowledged the added burden placed on their patients as a result of not being able to provide LARC methods on the same day as their initial visit. One staff highlighted the convenience factors associated with same-day insertions:

*“I think that would be great if we could do the same day, that’d be good…if you can get it all in one-stop shopping…” - Staff*

Interviewees mentioned the frustration of “losing people,” meaning patients not returning for their contraception at the appropriate time, usually several weeks after their initial visit. This was frustrating for several respondents:

*“[Making women come back for a second visit] is a barrier. I think we definitely lose people” - Provider*

Participants also supported the idea of on-site training in contraceptive counseling and LARC insertion, regardless of their position at the health center. One provider commented,

*“Now if we have some type of in-house training, then [providers and staff] all come. So they’ll get any educational benefit of training that we might get if there’s a speaker here or if there’s some information that is being done.” – Provider*

Several participants expressed an interest in training about contraception. One staff simply said,

*“I would love to have some [training].” - Staff*

### Barriers to implementing components of the CHOICE model

Despite overall support for the CHOICE model, participants described four system-level barriers to components of the CHOICE model; 1) limited insurance coverage and the cost of contraceptives, 2) scheduling challenges related to the same-day insertion of LARC methods, 3) requirements to order LARC devices from a third-party pharmacy for specific patients, and 4) specific health center policies and practices.

Cost and lack of insurance coverage were substantial barriers for this patient population, as noted by health center staff and providers. As some mentioned, patients might not be able to receive their choice of contraception if they do not have the ability to pay for it:

*“A lot of women ask for the pill, but once we tell them that [the program] won’t pay for it, then they’re like oh, well let me think about something else” - Staff*

One participant commented that lack of insurance prevents patients from coming to their clinic at all, serving as a barrier for any preventive services:

*“Individuals don’t come in for family planning if they don’t have insurance to pay. We see them once they get pregnant and they have no other choice but to come to us.” - Staff*

A second system-level barrier involves scheduling challenges to same-day insertion of LARCs. Participants expressed concern over the time involved in doing a LARC insertion, especially when it has not been scheduled upfront. One staff commented,

*“If they’re coming in for a well woman exam and then they’re here for an insertion…it’s the time factor…you can always rearrange that schedule but you’re cutting out time for other things, too, that is much needed.” Another staff discussed the difference between certain forms of birth control, “The LARC method it takes like 30 min, [DMPA shot] like 9 min. So it’s like time consuming.” - Staff*

Third, if LARC methods are required to be ordered by insurance companies before insertion, staff and providers described frustration with the ordering procedure. One staff specifically mentioned the time and work involved to obtain approval, order the device, and set up the secondary appointment:

*“First of all, if they have insurance, getting us to get their insurance card. Then I have to fax it. Then I have to wait for it to get approved…Then I call the patient back to…set up for an appointment. Then that’s how I think we end up losing them…Sometimes it can be a two to three week process.” - Staff*

If patients did not return for the insertion, many felt it was wasteful of both time and resources. Several participants commented on “being stuck” with the IUD or implant and not being able to use it for other patients:

*“We’ll order devices for patients and…I’ll try calling them and I’ll be like, “Okay we got your device in.” For whatever reason they don’t even have that number anymore, I can’t get a hold of them…So we’re just stuck with these devices.” - Staff*

*“You can only use it for that patient so it sit back there, you can’t use it for anybody else…Even if it’s expired, you have to “red-bag” it.” - Staff*

*“And it does get frustrating because we’re stuck with these devices…they’re just stacked up…they’re expensive…this is a lot of money that’s going down the drain.” - Staff*

Finally, participants mentioned health center practices that could discourage same-day insertion. One patient offered that she had to take a pregnancy test and still could not receive her chosen method that day:

*“I have to wait for two weeks…the protocol is that you take one pregnancy test. If it’s negative, come back in two weeks.” - Patient*

Providers and staff also discussed protocols and practices that served as potential barriers to implementing same-day insertions. For example, one provider said:

*“Well, the process, the way it works is that her first visit we’ll first talk about it and then she’ll get cultures. We’ll get cultures first. Then she’ll actually need to come back when she’s on a cycle, her monthly cycle.” - Provider*

Health center barriers extended beyond center protocols and requirements. Several participants indicated that the ability to perform same-day LARC insertions was based on provider guidelines or preference. Many providers and staff discussed this variability and inconsistency. One stakeholder shared that providers had preferred times of day when considering if or when to insert a LARC device:

*“If a patient want to come in and get [an implant] done, they can walk in and get it done but she only does it like eight o’clock in the morning.” - Staff*

In addition to these system-level barriers, participants described personal beliefs that might hinder the adoption of this model. These attitudes and values related to all aspects of the CHOICE model and were shared by providers, staff, and patients. First, providers indicated personal preferences for inserting IUDs in their patient populations that might be in conflict with the goal of same-day insertions. One provider stated:

*“We have a lot of STDs, so I do prefer to do cultures before I put in an IUD, so we’ll do those and then bring them back” - Provider*

Second, some participants held onto false information, even when given accurate data. Participants expressed notions about which types of patients were an appropriate candidate for certain types of contraception. As one said:

*“They say that the [DMPA shot] won’t hurt if the patient is pregnant but I just don’t believe it. I believe that if something can stop you from getting pregnant…I think it might cause some harm to the baby. That’s just my opinion.” - Staff*

Patients also described feeling like they might not be open to the CHOICE model, specifically the same-day insertion component. While some patients appreciated not coming back to the clinic 2-3 weeks later, some patients mentioned they would prefer more time to decide on a contraceptive method and might feel pressured by same-day insertion:

*“I don’t just make decisions on the fly. I have to get everyone else’s opinion and figure out what I want to do.” - Patient*

## Discussion

This qualitative research provides insight into how health center providers, staff, and patients approach a patient-centered model of contraceptive provision. By conducting qualitative interviews with key stakeholders based on a theoretical framework of implementation research, we were able to gain an understanding of system-, provider/staff-, and patient level reactions to implementing an evidence-based and patient-centered model of care. Our findings suggest that there is a limited understanding or knowledge about contraception among the health center community. Stakeholders would welcome additional information, via counseling or training, for patients and health center providers and staff. They also expressed support for providing same-day insertions of LARC methods. However, they identified several barriers to implementing components of the CHOICE model due to larger system-level barriers and personal beliefs. Our findings are similar to a prior study assessing contraceptive provision to teens and young women which found common challenges to providing contraceptive and LARC services were the costs of LARC methods, staff concerns about IUD use, and limited training on implant insertion [25].

These findings suggest that in addition to staff and provider training about contraceptive provision, implementation of the CHOICE model requires strategies to manage the patient and system-level barriers to receiving contraceptive methods. If results of the CHOICE model translate to real-world practice, a high number of patients might choose LARC methods requiring health centers to alter procedures to meet patients’ needs and desire for LARCs. For example, evidence-based guidelines recommend initiating IUDs and implants anytime that pregnancy can reasonably be ruled out [26] as the requirement for a second visit for insertion creates a patient barrier. In addition, to ensure adequate patient access, health centers must keep IUDs and implants stocked on the shelves and available for same-day insertion. However, many providers and staff described practices in their health centers that discouraged this approach. Addressing these system constraints could improve the likelihood that patients receive their chosen contraceptive methods.

After the passage of the ACA and implementation of the contraceptive coverage guarantee, many cost-related barriers to receiving contraception were removed. However, some insurance companies do not cover all methods, and current billing practices do not allow for reimbursement for contraceptive counseling provided by a non-clinician, a key component of the CHOICE model [5]. Health care centers could benefit from a more complete understanding of insurers’ rules and procedures, and how to properly bill for contraceptive services so that health centers are adequately reimbursed and patients are not erroneously charged for care [5]. In addition, payment reform could address the lack of reimbursement for contraceptive counseling provided by a trained health educator. Addressing out-of-pocket costs are key to facilitating patients’ desired contraceptive method and reducing unintended pregnancy [3, 4].

### Limitations

The findings should be interpreted in the context of this qualitative approach, which was not designed to provide a representative sample of responses, rather to highlight in-depth responses to CHOICE model components. In addition, the centers were all Federally Qualified Health Centers serving low-income, urban patient populations and therefore the results may not be transferable to other settings. Nonetheless, the strengths of this approach based on a well-established implementation research framework and the population studied allowed us to examine real-world challenges to implementation with direct input from patients, health center staff and clinicians.

## Conclusions

Overall, community health centers were willing and interested in a patient-centered model of contraceptive provision if key system-level barriers could be addressed. Addressing patient and clinician knowledge about effective contraception, attending to cost-related issues, and providing same-day insertion when possible can increase the uptake of highly effective contraceptives. Successfully planning for identified challenges to implementing the CHOICE model can improve the likelihood of broader adoption of the model, or adaptations of it, in real-world settings. Successful implementation can ultimately decrease unintended pregnancy in high-need populations.

## Abbreviations

ACA: Affordable care act
DMPA shot: Depot medroxyprogesterone acetate
FQHC: Federally qualified health center
IMPACCT: Innovative Model of PAtient-Centered ContracepTion study
IUD: Intrauterine device
LARC: Long-acting reversible contraception
